# A Retrospective Interventional Cohort Study to Assess the Safety and Efficacy of Sandostatin LAR for Treatment of Recurrent and/or Refractory Meningiomas

**DOI:** 10.3389/fneur.2020.00373

**Published:** 2020-05-06

**Authors:** Maya Hrachova, Emely Nhi T. Nguyen, Beverly D. Fu, Manisha J. Dandekar, Xiao-Tang Kong, Gilbert Cadena, Frank P. K. Hsu, John Billimek, Thomas H. Taylor, Daniela A. Bota

**Affiliations:** ^1^Department of Neurology, Irvine Medical Center, University of California, Orange, Orange, CA, United States; ^2^School of Medicine, University of California, Irvine, Irvine, CA, United States; ^3^Chao Family Comprehensive Cancer Center, Irvine Medical Center, University of California, Orange, Orange, CA, United States; ^4^Department of Neurological Surgery, Irvine Medical Center, University of California, Orange, Orange, CA, United States; ^5^Department of Epidemiology, University of California, Irvine, Irvine, CA, United States

**Keywords:** recurrent progressive meningioma, Somatostatin LAR, octreotide, skull based meningioma, meningioma size, meningioma surgery

## Abstract

**Background:** Meningiomas are the most common adult primary intracranial tumors in the United States. Despite high recurrence rate of atypical and malignant subtypes, there is no approved drug indicated specifically for meningioma. Since the majority of meningiomas exhibit high density of somatostatin receptors subtypes, somatostatin analogs have been under close investigation. The aim of this study was to evaluate efficacy and safety of Sandostatin LAR (octreotide) in patients with progressive, and/or recurrent meningioma, and identify subset of patients who were more likely to benefit from this treatment.

**Methods:** A total of 43 patients ≥ 18 years old were included in the retrospective chart review. The patients underwent treatment with Sandostatin LAR (octreotide) from 01.01.2010 to 06.01.2017 at the University of California, Irvine after confirmation of the diagnosis. Six months progression free survival (PFS6) was defined as a primary endpoint, and the overall survival (OS), safety, and toxicity were identified as secondary endpoints.

**Results:** The OS for 6 months, 1, and 3 years for all WHO grades was 94.8, 88.1, and 67.0%, respectively. The PFS6 for WHO I, II, III, and all was 89.4, 89, 33.3, and 80% respectively. For patients with no prior surgeries, chemotherapy or radiation, the PFS6 was 88.9, 84.8, and 94.8%, respectively. Interestingly, the PFS6 was 90.5% for skull-based and 80% for 3–6 cm tumors. Patients with tumors in parasagittal location had PFS6 of 83.3% compared to PFS6 of 50.0% for patients with convexity tumors. Evaluation of PFS6 based on the effect of estrogen and progesterone on meningioma identified that ER-PR+ tumors had PFS6 of 87.8% while patients with ER-PR- meningiomas had PFS6 of 62.5%. Median TTP for WHO grade I, II, and III was 3.1, 2.40, and 0.26 years, respectively. Subgroup analysis showed that median TTP was 3.1 years for <3 cm tumors, 3.22 years for skull-based tumors, 2.37 years for patients with prior surgeries and 3.10 years for patients with no history of chemotherapy. History of radiation had no effect on median TTP. Sandostatin LAR (octreotide) was well-tolerated.

**Conclusions:**This is one of the largest retrospective analysis of meningioma patients treated with Sandostatin LAR (octreotide) suggesting that this treatment has minimal to no adverse events and could prolong overall survival, and progression free survival especially for patients with ER-PR+ tumors who underwent surgeries for small skull-based tumors.

## Introduction

For Meningiomas are dural-based tumors that arise from an arachnoid layer or meningothelial cells. They are the most common primary adult CNS tumors, and account for 36.8% of all primary brain tumors (1). Most meningiomas are histologically classified as World Health Organization (WHO) grade I tumors (benign, 81.3%) with an indolent course. WHO grade II (atypical, 16.9%) and WHO grade III (anaplastic, 1.7%) tumors classified as high-grade tumors and known to be more aggressive with increased risk of recurrence (2).

If indicated based on tumor size or tumor progression, patients with WHO grade I meningiomas undergo complete surgical resection (3). Approximately 5% of completely resected benign meningiomas, 30% of partially resected benign meningiomas and 40% of atypical meningiomas recur within 5 years after surgery (4). Despite surgical resection and radiation therapy that is the standard of care for WHO grade II and III meningiomas, patients have higher recurrence risk of 29–52 and 50–94%, respectively (5). Depending on tumor location, invasion of surrounding structures, age, and medical comorbidities of the patient, surgical intervention is not always possible. Chemotherapy or biologics are then considered as an alternate treatment option. There is no FDA approved drug indicated specifically for meningioma, and patients with atypical, anaplastic, recurrent, or invasive meningiomas are often left with limited options.

The National Comprehensive Cancer Network (NCCN) guideline identified three drug classes that showed some benefits for treatment of meningioma in retrospective analysis or small phase II trials: vascular endothelial growth factor (VEGF) signaling pathway inhibitors, alpha-interferons, and somatostatin receptor agonists (6, 7). Since the majority of meningiomas exhibit a high density (70%) of somatostatin receptors subtypes (SSTR1–SSTR5), it is not surprising that somatostatin analogs have been under close investigation as a potential treatment option (8, 9).

Somatostatin is an acyclic tetradecapeptide hormone that is produced in hypothalamus and released into systemic circulation, where it exhibits its exocrine and endocrine inhibitory functions by targeting pituitary, pancreas and gastrointestinal tract (10). It also has been implicated in the induction of apoptosis and inhibition of angiogenesis (11). Since naturally occurring somatostatin has a short half-life, somatostatin analogs were developed to achieve a longer half-life (lanreotide, pasireotide, and octreotide).

Sandostatin LAR (octreotide) is another long acting somatostatin analog approved by the FDA for treatment of acromegaly, severe diarrhea/flashing episodes associated with metastatic carcinoid tumors, and vasoactive intestinal peptide (VIP) secreting tumors (12).

Numerous *in vitro* studies investigated antitumor effect of octreotide acetate. For instance, Arena et al. evaluated a role of SSTR in the control of human meningioma cell proliferation and identified that in four out of six primary cell cultures obtained from fresh meningioma surgical sample, the treatment with somatostatin caused inhibition of DNA synthesis induced by the tumor-promoter phorbol myristate acetate (13). Graillon et al. investigated the signal transduction pathways triggered by octreotide and correlated inhibition to cellular markers using a large set of all histological subtypes of meningioma (14). Study showed that octreotide significantly decreased cell proliferation in 88% of meningiomas but did not induce cell death. It was postulated that it had an effect on the level of phosphorylated p70-S6 kinase implicated in rapamycin (mTOR) pathway.

Given that Sandostatin LAR (octreotide) was shown to significantly decrease cell proliferation in 88% of meningiomas with more prominent inhibition in a group expressing a high level of SSTR2a, one of the most frequently expressed receptors in meningiomas, investigative work was initiated to assess its efficacy for treatment of meningiomas (8, 15).

A prospective pilot study showed that 31% of patients with recurrent meningiomas demonstrated a partial radiographic response and 44% achieved progression free survival (PFS) at 6 months with minimal side effects after undergoing treatments with Sandostatin LAR (octreotide) (16). Even though a phase II study conducted to evaluate the efficacy and safety of Sandostatin LAR (octreotide) did not demonstrate a significant benefit, 2 patients experienced prolonged stability of previously progressive tumors (17). Studies that investigated the effect of Sandostatin LAR (octreotide) in patients with a progressive benign residual or recurrent meningioma of the skull base, showed that somatostatin analog can arrest progression and stabilized disease (14).

Numerous clinical studies highlighted potential benefit of Sandostatin LAR (octreotide) for treatment of meningioma, but due to small sample size, no statistical significance was achieved. Thus, our retrospective interventional cohort study with a bigger sample size provides supporting evidence to consider Sandostatin LAR (octreotide) as a potential candidate for meningioma-based treatment taken into an account its tolerability and safety profile.

## Materials and Methods

The following study was a retrospective interventional cohort analysis conducted at the University of California Medical Center (UCIMC) between January 2010 and June 2017. The study cohort consisted of patients with recurrent and/or progressive WHO grade I, II, or III meningiomas who received treatment with Sandostatin LAR (octreotide). All information related to patients' demographics, cancer type, response to treatment, therapies previously received, and Karnofsky performance scores (KPS) were collected (18). The primary objective of this study was to determine efficacy of Sandostatin LAR (octreotide) in patients with recurrent and/or progressive meningiomas. Six months PFS6 was defined as a primary endpoint, and OS was a secondary endpoint. Safety and toxicity of Sandostatin LAR (octreotide) were assessed as well (19).

### Patients Eligibility

Patients were required to be ≥ 18 years old with recurrent and/or progressive meningioma expressing sandostatin receptors confirmed by positive ^111^Indium (^111^In)—octreotide positron emission tomography (PET) and/or positive immunohistochemistry analysis. The majority of the patients (38) had positive PET scan while remaining patients (5) were diagnosed based on the pathology results. Histological typing and grading of tumors according to the WHO grading system were done via hematoxin and eosin staining. Immunohistochemical staining were done for estrogen receptor (ER), progesterone receptor (PR), Ki-67 and Sandostatin based on the University of California of Irvine protocol. Patients were determined to be poor candidates for surgical resection, stereotactic radiosurgery, or radiation therapy based on tumor location, increased risk factors for postoperative morbidity and mortality, or individual preference for non-invasive approach, or were shown to have recurrence despite surgical or radiation therapy. Patients who suffered from meningioma recurrence were offered treatment independent of history of prior surgeries, chemotherapy, radiation, or radiosurgery treatments. Patients were excluded if metastatic lesions were found on octreotide PET scan or informed consent was not obtained.

### Tumor Variables

Tumor size and location were obtained from the MRI scan and official radiologist's report. The largest diameter was used as an overall surrogate for tumor size. Tumor size was categorized into 3 groups: small (tumor <3.0 cm in diameter), medium (tumor ranging 3–6 cm in diameter) and large (tumor more than 6 cm in diameter). Tumor location was subdivided into 3 groups: skull based (cavernous sinus, cerebellopontine angle, clinoid, clivus, foramen magnum, jugular foramen, middle fossa, olfactory groove, orbital, parasellar, petro-clival, petrous, planum sphenoidale, posterior fossa, skull base, sphenoid wing, and tuberculum sellae), falx/parasagittal/convexity, and mixed.

### Treatment Plan

The diagnosis of meningioma was confirmed either via ^111^In-octreotide PET scan or by immunohistochemical analysis. Imaging study (MRI or CT) was done prior to the first drug administration and was repeated every 2–3 months afterwards for an evaluation of tumor status. Imaging studies were used to define the disease recurrence. Patients received deep intragluteal injections of Sandostatin LAR (octreotide) monthly and were treated until disease progression or intolerability. The dose of Sandostatin LAR (octreotide) was gradually increased from 30 to 40 mg per injection if tolerated. Patients were followed for any adverse reactions to the drug. The treatment was stopped if the patient met any of the following criteria: MRI or CT showed tumor progression, serious adverse events, physician discretion, patient's choice to discontinue treatment, death, or lost to follow up. The institutional review board approved the study, and all patients that participated provided written informed consent.

### Statistical Methods

Data was analyzed using the IBM SPSS statistical program package (PAWS statistics v18.0). Data was grouped into categories based on demographics, WHO tumor grades, KPS scores, tumor and treatment characteristics, and analyzed using descriptive statistics. The PFS was calculated from the date of initial treatment with Sandostatin LAR (octreotide) until the date of death or disease progression. Patients who did not experience disease progression were censored. The OS was estimated from the date of initial treatment with Sandostatin LAR (octreotide) to the date of death or last known date to be alive. Subjects that have not died were censored at the last known date to be alive. Survival curves were estimated by generating Kaplan-Meier methods. PFS and OS were compared between WHO tumor grades, tumor, and treatment characteristics. The log rank test was used to compare the survival distributions of the groups. *P* < 0.05 for all analyses was considered significant. A proportional-hazards model was used to delineate the risk of death adjusted for covariates. Best radiographic response was determined based on 2010 the Response Assessment in Neuro-Oncology (RANO) Working Group (20). Results from our treatment group were compared to results from previous published studies using Sandostatin LAR (octreotide) for treatment of meningioma.

### Safety and Toxicity

Adverse events were reported by patients and/or providers when abnormal laboratory or physical examination findings were identified requiring intervention. Adverse events were recorded from the first date of Sandostatin LAR (octreotide) administration until death or 12 months follow up. The relationship of the adverse event to Sandostatin LAR (octreotide) was also evaluated. It was considered to be a related event when there was an evidence to suggest the relationship between the drug and the adverse event. An unrelated event was thought to be an adverse event, possibly caused by an underlying disease or biologically improbable event. Safety results were evaluated via descriptive statistics to identify frequency, type, and severity of adverse events.

Treatment related toxicities were evaluated using the Common Terminology Criteria for Adverse Events (CTCAE) version 4.03 (21). All patients who received Sandostatin LAR (octreotide) were evaluable for toxicity, and toxicity results were compared to other clinical studies.

## Results

### Patients Characteristics

A total of 43 patients with recurrent or progressive WHO grade I (75.0%), II (11.4%), and III (13.6%) meningiomas were enrolled in this study, including 5 with atypical and 6 with anaplastic meningiomas (Table 1). The majority of patients were females (70.5%) who identified as White (38.6%), Hispanic (25.0%), or Asian (22.7%). Median age was 65 years old. Median KPS score was 80. Median number of Sandostatin LAR (octreotide) injections was 8. Evaluation of prior treatments identified that 75.0% of patients with all tumor grades had surgical resections, 45.4% had radiation therapy while 13.6% underwent chemotherapy. Analysis of prior recurrences identified 12 patients with WHO grade I tumors who had no prior recurrences, 7 patients with one recurrence, 10 patients with two recurrences, and 4 patients with three or more recurrences. All patients with WHO grade II and III meningiomas were noted to have two or more recurrences. The cohort consisted of small (<3.0 cm) and medium (3–6 cm) tumors that were predominantly skull base tumors (23 patients). We identified 25 patients with ER positive (ER+), one patient with ER negative (ER-), 18 patients with PR positive (PR+), and 8 patients with PR negative (PR-) statuses. Subgroup analysis showed 17 patients with ER+PR+, 8 patients with ER-PR- and one patient with ER-PR- statuses.

**Table 1:** Patient demographic and clinical characteristics (*n* = 43).

**Characteristics**	**All Patients**			
Median Age (years) (range)	66 (35–90)			
Male, No. (%)	13 (29.5)			
Female, No. (%)	30(70.5)			
**Ethnicity/race, no. (%)**				
•White •Hispanic •Asian •Other	16 (37.2) 11 (25.6) 9 (20.9) 7 (16.3)			
Median number of Sandostatin LAR injections	8 treatments (1–25)			
**KPS score at baseline, no. (%)**				
•50 •60 •70 •80 •90 •100 •Median	1 (2.3) 2 (4.5) 9 (20.5) 15 (34.1) 13 (29.5) 3 (7) 80			
**WHO tumor grade no. (%)**				
•1 •2 •3	32 (74.4) 5 (11.6) 6 (13.9)			
	**WHO Grade 1**	**WHO Grade 2**	**WHO Grade 3**	**All Grades**
**Prior treatments no. (%)**				
•Resection •Chemotherapy •Radiation therapy	24 (55.8) 3 (7.0) 12 (30.0)	5 (11.6) 1 (2.3) 2 (4.7)	5 (11.6) 3 (7.0) 6 (14.0)	34 (79.1) 7 (16.3) 20 (46.5)
**Previous recurrences no. (%)**				
•0 •1 •2 •3 <	11 (25.6) 7 (16.9) 10 (23.3) 4 (9.3)	0 (0) 0 (0) 3(7.0) 2 (4.7)	0 (0) 0 (0) 2 (4.7) 4 (9.3)	11 (25.6) 7 (16.9) 15 (34.8) 10 (23.3)
**Tumor size no. (%)**				
•Small (<3.0 cm) •Medium (3–6 cm) •Large (> 6 cm)	17 (39.5) 14 (32.6) 1 (2.3)	1 (2.3) 3 (7.0) 1 (2.3)	2 (4.7) 4 (9.3) 0	20 (46.5) 21 (51.2) 2 (4.7)
**Tumor location no. (%)**				
•Skull base •Parasagittal •Convexity/Falx •Mixed	21 (48.8) 3 (7) 3 (7) 5 (11.6)	1 (2.3) 1 (2.3) 3 (7) 0	1(2.3) 2 (4.7) 3 (7) 0	23 (53.5) 6 (34.8) 9 (20.9) 5 (11.6)

### Toxicity

Toxicity data is reported for all 43 patients (Table 2). In general, therapy with Sandostatin LAR (octreotide) was well-tolerated. No CTCAE grade 4 or 5 adverse events were observed. Sandostatin LAR (octreotide) treatments were discontinued in two patients after they experienced significant adverse events. One patient developed cholelitiasis complicated by pancreatitis identified as grade 3 adverse event. The other patient experienced vomiting that was defined as grade 2 event. The majority of grade 2 adverse events included diarrhea (11.4%), headache (6.8%), nausea (4.5%), and abdominal pain (4.5%). Patients more frequently experienced grade 1 events with diarrhea (27.3%) and headache (27.3%) being most common side effects.

**Table 2:** Treatment related CTCAE adverse events (*n* = 43).

**Adverse events**	**CTCAE Grade 1 No. (%)**	**CTCAE Grade 2 No. (%)**	**CTCAE Grade 3 No. (%)**	**CTCAE Grade 4 No. (%)**	**CTCAE Grade 5 No. (%)**	**Total No. (%)**
Diarrhea	12 (28)	4 (9.3)	0	0	0	16 (37.2)
Loose stools	5 (11.6)	0	0	0	0	5 (11.6)
Headache	11 (25.6)	3 (7.0)	0	0	0	14 (32.6)
Local pain	6 (14.0)	0	0	0	0	6 (14.0)
Flu like symptoms	3 (7.0)	1 (2.3)	0	0	0	4 (9.3)
Weakness	1 (2.3)	1 (2.3)	0	0	0	2 (4.7)
Palmar redness	1 (2.3)	0	0	0	0	1 (2.3)
Chills	1 (2.3)	0	0	0	0	1 (2.3)
Sweats	1 (2.3)	0	0	0	0	1 (2.3)
Arthralgia	1 (2.3)	0	0	0	0	1 (2.3)
Nausea/vomiting	3 (7.0)	2 (4.7)	0	0	0	5 (11.6)
Abdominal pain	2 (4.7)	2 (4.7)	0	0	0	4 (9.3)
Insomnia	3 (7.0)	1 (2.3)	0	0	0	4 (9.3)
Dizziness	4 (9.3)	0	0	0	0	4 (9.3)
Constipation	9 (20.9)	1 (2.3)	0	0	0	10 (23.3)
Anxiety	0 (0)	1 (2.3)	0	0	0	1 (2.3)
Fatigue	4 (9.3)	0	0	0	0	4 (9.3)
Pancreatitis	0	0	1 (2.3)	0	0	1 (2.3)
Cholelithiasis	0	0	1 (2.3)	0	0	1 (2.3)
Abdominal bloating	1 (2.3)	0	0	0	0	1 (2.3)

### Response and Outcome

**Figure 1:**
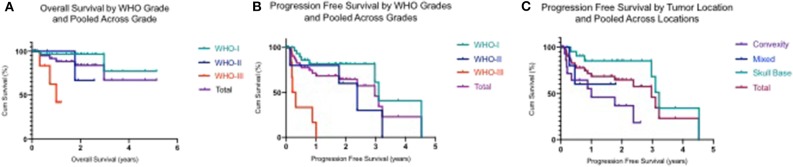
**(A)** Kaplan–Meier plot demonstrating overall survival by WHO tumor grades and polled across WHO grades. **(B)** Kaplan–Meier plot demonstrating progression free survival by WHO tumor grades and polled across WHO grades. **(C)** Kaplan–Meier plot demonstrating progression free survival by tumor location and polled across locations.

Analysis indicated that the median PFS for all tumor grades was 3.0 years (95% CI: 2.20–3.80), PFS6 80.6% (95% CI: 0.68–0.93), PFS12 71.9% (95% CI: 0.58–0.86), and PSF36 46.2% (95% CI: 0.21–0.72) (Table 3.1). The median PFS for WHO grade I meningiomas was 3.1 years (95% CI: 2.80–3.40), PFS6 89.8% (95% CI: 0.79–1.00), PFS12 82.0% (95% CI: 0.67–0.97), and PSF36 61.5% (95% CI: 0.25–0.98). The median PFS for WHO grade II meningiomas was 2.4 years (95% CI: 1.40–3.30), PFS6 80.0% (95% CI: 0.45–1.00), PFS12 80.0% (95% CI: 0.45–1.00), and PSF36 30.00% (95% CI: 0.00–0.77). The median PFS for WHO grade III meningiomas was 0.2 years (95% CI: 0.05–0.36), patients in this group did not survive past 6 months. The log rank test had a value of *p* < 0.001 which means that there was a statistically significant difference in PFS between the WHO tumor grade groups. Kaplan-Meier curves were generated to analyze the overall PFS and PFS stratified by WHO tumor grade (Figures 1A,B). Evaluation based on tumor location showed that PFS6 was 90.5% (CI: 0.79–1.00) for skull base tumors with 83.3% (CI: 0.54–1.00) for parasagittal meningiomas while PFS6 was 50.0% (0.15–0.85) for convexity lesions (Table 3.2). Median PFS for small tumors was 3.22 years (CI: 2.96–4.53) (Table 3.2). Analysis based on the treatment history identified that PFS6 for patients with no history of radiation was 94.7% (CI: 0.85–1.00), no surgeries 88.9% (CI: 0.68–1.00) and no chemotherapy was 84.8% (CI: 0.73–0.97) (Table 3.3). Patients with ER-PR+ tumors had PFS6 of 87.8% (CI: 0.72–1.00) while patients with ER-PR- meningiomas had PFS6 of 62.5% (CI: 0.28–0.96).

**Table 3.1:** Median progression free survival and progression free survival at 6 months, 1 year, and 3 years (*n* = 43).

**PFS**	**WHO Grade 1 (CI 95%)**	**WHO Grade 2 (CI 95%)**	**WHO Grade 3 (CI 95%)**	**ALL (CI 95%)**
6 months	89.4% (0.78–1.00)	80.0% (0.45–1.00)	33.3% (0.00–0.71)	80% (0.67–0.92)
1 year	81.5% (0.67–0.96)	80.0% (0.45–1.00)	–	68.3% (0.53–0.83)
3 years	61.1% (0.25–0.97)	30.0% (0.00–0.77)	–	45.9% (0.20–0.71)
**Median PFS**				
Median	3.1 years	2.4 years	0.2614 years	2.97 years
	(3.0–4.5)	(1.3–3.2)	(0.17–1.0)	(1.76–4.53)

*(–), undetermined*.

**Table 3.2:** Median progression free survival and progression free survival at 6 months, 1 year, and 3 years based on tumor location and size (*n* = 43).

**PFS**	**Parasagittal (CI 95%)**	**Convexity (CI 95%)**	**Skull Base (CI 95%)**	**Mixed (CI 95%)**	**Size (<3 cm) (CI 95%)**	**Size (3-6 cm) (CI 95%)**	**Size (>6 cm) (CI 95%)**
6 months	83.3% (0.54–1.00)	50.0% (0.15–0.85)	90.5% (0.79–1.00)	60% (0.17–1.00)	73% (0.54–0.92)	80% (0.63–0.98)	100
1 year	62.5% (0.20–1.00)	33.3% (0–0.68)	85.2% (0.70–1.00)	–	66.4% (0.44–0.89)	68.7% (0.48–0.90)	–
3 years	–	–	68.1% (0.36–1.00)	–	44.3% (0.06–0.83)	22.9 (0–0.60)	–
Median PSF	2.38	0.68	3.22 (2.96–4.53)	–	3.10 (0.51–4.53)	2.38 (0.87–3.22)	1.77 (–)

*(–), undetermined*.

**Table 3.3:** Median progression free survival and progression free survival based on treatment history at 6 months, 1, and 3 years (*n* = 43).

**PFS**	**Radiation (CI 95%)**	**Non-radiation (CI 95%)**	**Surgical (CI 95%)**	**Non-surgical (CI 95%)**	**Chemo-therapy (CI 95%)**	**Non-chemotherapy (CI 95%)**
6 months	85.2% (0.70–1.00)	94.7% (0.85–1.00)	74.3% (0.59–0.90)	88.9% (0.68–1.00)	42.9% (0.06–0.80)	84.8% (0.73–0.97)
1 year	85.2% (0.70–1.00)	94.7% (0.85–1.00)	62.7% (0.45–0.80)	–	14.3% (0–0.40)	81.0% (0.67–0.95)
3 years	49.7% (0.07–0.93)	82.9% (0.59–1.00)	37.4% (0.10–0.64)	–	–	57.6% (0.27–0.88)
Median PFS	2.97 (1.76–(–))	2.97 (1.76–(–))	2.37 years (0.87–4.53)	–	0.51 (0.19–1.0)	3.10 (2.37–4.53)

*(–), undetermined*.

The median OS for all tumor grades has not been yet reached, thus it could not be reported (Table 4.1). There was a low event rate in which half of patients remained alive. The median OS for WHO grade III meningioma was 1.0 (95% CI: 0.45–1.56). The OS for all tumor grades at 6 months was 94.9% (95% CI: 0.88–1.00), 88.4% (95% CI: 0.78–0.99) at 1 year, and 67.2% (95% CI: 0.36–0.99) at 3 years. The OS was also calculated for each WHO tumor grade at 6 months, 1 and 3 years. The OS for I, II, and III at 6 months were 96.6% (95% CI: 0.90–1.00), 100%, and 83.3% (95% CI: 0.54–1.00) respectively. At 1 year, the OS for WHO grade I was 96.6% (95% CI: 0.90–1.00), 100% for WHO grade II and for WHO grade III was 62.5% (95% CI: 0.21–1.00). The OS at 3 years for WHO grade I was 77.2% (95% CI: 0.43–1.00). Kaplan Meier curves were generated to show OS and OS stratified by WHO grade (Figures 1C,D). The median OS was achieved for patients with convexity tumors (1.75 years), medium tumor size (2.97 years), and 2.97 years for patients with no prior history of radiation or surgeries (Tables 4.2 and 4.3).

**Table 4.1:** Median overall survival and overall survival at 6 months, 1, and 3 years (*n* = 43).

**OS**	**WHO Grade 1 (CI 95%)**	**WHO Grade 2 (CI 95%)**	**WHO Grade 3 (CI 95%)**	**ALL (CI 95%)**
6 months	96.4% (0.90–1.00)	100%	83.3% (0.54–1.00)	94.8% (0.85–1.00)
1 year	96.4% (0.90–1.00)	100%	41.7% (0.00–0.85)	88.1% (0.77–0.99)
3 years	77.1 (0.43–1.00)	*	*	67.0% (0.36–0.98)
Median OS	–	–	1.0 (0.31–(–))	–

*Final case was censored before this point, (–), undetermined.

**Table 4.2:** Median overall survival and overall survival at 6 months, 1 year, and 3 years based on tumor location and size (*n* = 43).

**OS**	**Parasagittal (CI 95%)**	**Convexity (CI 95%)**	**Skull Base (CI 95%)**	**Mixed (CI 95%)**	**Size (<3 cm) (CI 95%)**	**Size (3-6 cm) (CI 95%)**	**Size (>6 cm) (CI 95%)**
6 months	100%	87.5% (0.64–1.00)	95% (0.85–1.00)	–	94.4% (0.84–1.00)	94.7% (0.85–1.00)	100%
1 year	80% (0.44–1.00)	65.6% (0.24–1.00)	95% (0.85–1.00)	–	–	82.1% (63.6–100)	–
3 years	–	–	76% (0.42–1.00)	–	–	–	–
Median OS	–	1.75 (–)	–	–	–	2.97 (–)	

*(–), undetermined*.

**Table 4.3:** Median overall survival and overall survival based on treatment history at 6 months, 1 year, and 3 years (*n* = 43).

**OS**	**Radiation (CI 95%)**	**Non-radiation (CI 95%)**	**Surgical (CI 95%)**	**Non-surgical (CI 95%)**	**Chemo-therapy (CI 95%)**	**Non-chemotherapy (CI 95%)**
6 months	95% (0.85–1.00)	94.7% (0.85–1.00)	96.7% (0.90–1.00)	88.9% (0.68–1.00)	100%	93.7% (0.85–1.00)
1 year	83.1% (0.66–1.00)	94.7% (0.85–1.00)	88.1% (0.75–1.00)	88.9% (0.68–1.00)	83.3% (0.54–1.00)	89.6% (0.78–1.00)
3 years	83.1% (0.66–1.00)	41.5% (0–1.00)	82.6% (0.66–0.98)	0	62.5% (0.20–1.00)	67.2% (0.28–1.00)
Median	–	2.97 (1.76–(–))	–	2.97 (0.35–2.97)	–	–

*(–), undetermined*.

## Discussion

Currently, there are no FDA approved therapies for management of surgically inaccessible or radiation-refractory recurrent meningiomas. In 2019, the Central Nervous System NCCN guideline recommended somatostatin analogs as valuable therapeutic options for management of progressive or recurrent meningiomas (6). These recommendations were derived from limited studies evaluating various somatostatin analogs (somatostatin, pasireotide, octreotide, and Sandostatin LAR). Our study, on the other hand, is one of the largest reported retrospective analysis of meningioma patients with recurrent and/or progressive disease treated with Sandostatin LAR (octreotide).

Comprehensive review of our study and previously reported data are summarized in Table 5. We utilized similar inclusions and exclusions criteria as prior trials. For instance, we included adult patients with recurrent or progressive meningioma diagnosed by PET scan (14, 22) and/or biopsy proven as in Johnson et al. (17). Prior radiotherapy, chemotherapy treatments and/or surgeries were permitted, as it was done in Chamberlain et al. and Johnson et al. (16, 17), in contrast to the study by Simo et al. that included only chemotherapy naïve patients (22). The results of our study were compatible and showed noted improvement from prior investigations. Specifically, PFS6 in our study for all tumor grades was 80.0% while prior studies reported PFS6 in 32.0–44.4% range (14, 16, 17, 22).

**Table 5:** Studies of octreotide analogs in refractory recurrent meningiomas.

**Author, year of publication**	**Number of patients and WHO grade of meningioma**	**Median KPS**	**PFS6 (%)**	**Median TTP/PFS (months)**	**Median OS (months)**	**Common toxicities**
Chamberlain et al. Sandostatin LAR (16)	16 (I, *n* = 8; II, *n* = 3; III, *n* = 5)	80	44	5	7.5	Diarrhea
Johnson et al. Sandostatin LAR (17)	Meningiom a:11 (I, *n* = 3; II, *n* = 3; III, *n* = 5) Hemangiopericytom a: 1	ND	ND	4.25	32.4	Diarrhea, anorexia, nausea, transaminitis
Schulz et al. Sandostatin LAR (14)	13 (I, *n* = 8 localized to skull base that underwent analysis)	ND	ND	24	ND	Well-tolerated, *n* = 1 psychiatric side effects
Simo et al. Octreotide (22)	9 (II, *n* = 5; III, *n* = 4)	80	44.4	4.23	18.7	Diarrhea
Norden et al. Pasireotide LAR (SOM230C) (23)	28 (I, *n* = 16; II/III *n* = 18)	85	32	4.5	Not achieved	Hyperglycemia, hypoglycemia, elevated amylase, elevated lipase, fatigue, hypokalemia
Graillon et al. Everolimus and Octreotide (24)	20 (I, *n* = 2; II, *n* = 10, III *n* = 8)	ND	55	ND	ND	Stomatitis, fatigue, diarrhea
Our study	43 (I, *n* = 32; II, *n* = 5, III *n* = 6)	80	80	35.6	Not achieved	Diarrhea, headache

The OS for all tumor grades has not been achieved in our study, while other investigations reported OS ranged from 7.5 to 34.2 months. Median KPS status in all studies ranged from 80 to 85. The prospective pilot trial conducted by Chamberlain et al. included 16 patients with low and high grade recurrent meningiomas who received treatment with Sandostatin LAR (octreotide) (16). Analysis indicated PFS6 of 44%, median TTP of 5 months and median OS of 7.5 months with minimal toxicity. The phase II study by Johnson et al. evaluated 12 patients with all tumor grades reported median TTP as 4.25 months and median OS as 32.4 months (17). Schulz et al. evaluated 13 patients with WHO tumor grade I meningiomas, and only median TTP was reported as 24 months (14). Simo et al. conducted phase II trial on patients with high-grade meningiomas who received subcutaneous octreotide injections every 28 days and reported PFS6 as 44.4%, median TTP as 4.23 months, and median OS as 18.7 months (22). Norden et al. showed no radiographic response to therapy with pasireotide LAR (SOM230C) on 28 patients with recurrent meningiomas of all tumor grades, PFS6 as 32% and median TTP as 4.5 months, but overall OS was not reached (23). The prospective phase II clinical trial by Graillon et al. evaluated the benefits of combination of everolimus and octreotide in patients with recurrent meningioma who were ineligible for further interventions (24). The PFS at 6 months was 55%, the overall survival rates at 6 and 12 months were 90 and 75%, respectively (24).

Only one prior study by Schulz et al. evaluated PFS based on the tumor location. Schulz et al. evaluated eight patients with skull based WHO grade I tumors and showed PFS was 100% at 48 months with two patients discontinued treatment after 36 months without disease progression (14). Our study included 23 patients with skull-based tumor of which 21 patients had WHO grade 1 meningiomas. The PFS6 for skull-based tumors was 90.5% which is consistent with Schulz et al. No analysis based on tumor grade, location or receptor type were performed in other studies that investigated Sandostatin LAR (16, 17, 22). Our data showed no radiological regression as defined by the RANO criteria (25) which is in agreement with previously published data. Based on *in vitro* studies showing that octreotide significantly decreased cell proliferation but did not induce cell death (13, 14), it is not surprising that prior prospective studies investigating the efficacy of Sandostatin LAR on meningioma showed no evidence of radiological tumor regression (14, 16, 17, 22, 23). It was suggested that even though no radiographical tumor regression was detected, Sandostatin LAR may arrest tumor progression (17). Only recent Phase II clinical trial that evaluated the effectiveness of combination of everolimus and octreotide showed that among the 20 study patients, radiological regression in the tumor volume by >10% was identified in 4 patients (24).

Evaluation based on tumor size and location revealed that the patients with skull-based and small tumors had the longest median PFS of 3.22 and 3.1 years respectively. Interestingly, our cohort showed no difference in the median PFS based on the radiation status while the patients with no history of chemotherapy were noted to have the median PFS of 3.10 years. The patients who underwent surgeries had the median PFS of 2.37 years. Thus, patients with small skull-based tumors with prior surgeries and no history of chemotherapy had the longest median PFS without respect to prior history of radiation.

Evaluation of PFS6 based on the effect of estrogen and progesterone on meningioma was performed. Analysis showed that patients with ER-PR+ tumors had PFS6 of 87.8% (CI: 87.8% (0.72–1.00) while patients with ER-PR- meningiomas had PFS6 of 62.5% (CI:0.28–0.96). These findings are in agreement with previously published data noting that lack of PR expression to be correlated with high tumor grade and tumor recurrence. Pravdenkova et al. showed that expression of the PR alone in meningioma signals a favorable clinical and biological outcome while the lack of receptors correlates with aggressive clinical behavior, progression or recurrence of this tumors (26). Recent retrospective study showed that patients with meningioma with ER+ had a much worse prognosis than those with ER weak or ER- status [Hua]. Since our analysis mainly included patients with ER+ status at least indicates that more than 58% of the cohort had ER+ status (27).

Reported toxicities were consistent across clinical studies with diarrhea, abdominal pain, nausea, vomiting and transaminities being the most common events. Prior studies had <25 patients that resulted in low statistical power.

Our clinical study provides additional evidence to support the rationale for a larger phase study to assess the efficacy of Sandostatin LAR (octreotide). Compared to other therapeutics, Sandostatin LAR (octreotide) had the longer median PFS, PFS6 and safety profile (Table 6). There were no observed CTCAE grade 4 or grade 5 adverse events. There was only one CTCAE grade 3 adverse event that identified the patient who was hospitalized with pancreatitis after developing cholelithiasis. Sandostatin LAR (octreotide) was subsequently discontinued in this patient. Erlotinib demonstrated a favorable safety profile compared to all the other drugs, however, median PFS was shorter than for the patients who received Sandostatin LAR (octreotide).

**Table 6:** Targeted therapy for progressive recurrent meningiomas.

**Author, year of publication**	**Inhibitor**	**Target**	**N**	**Tumor Grade**	**Median KPS**	**PFS6 (%)**	**Median TTP/PFS (months)**	**Common toxicities**	**Grade 4 or 5 toxicity**
Nayak et al. (28)	Bevacizumab	VEGF	15	II, III	ND	43.7	6.5	Fatigue, cerebral hemorrhage	No
Lou et al. (29)	Bevacizumab	VEGF	14	I, II, III	80	85.7	17.9	Thrombocytopenia, proteinuria, craniotomy site cellulitis	Yes
Nunes et al. (30)	Bevacizumab	VEGF	15	NF2	ND	85	15	Hypertension, transaminitis, menorrhagia, irregular menses	No
Alanin et al. (31)	Bevacizumab	VEGF	7	NF2	ND	ND	ND	Intracerebral hemorrhage	Yes
Shih et al. (32)	Bevacizumab+everolimus	VEGF mTOR	17	I, II, III	ND	69	22	Colitis, chronic thrombotic microangiopathy, proteinuria, nephrotic syndrome	No
Wen et al. (33)	Imatinib	PDGFR	23	I, II, III	80	29.4	2	Anemia, leukopenia, neutropenia, dehydration, dizziness, hypophosphatemia	Yes
Horak et al. (34)	Imatinib	PDGFR	9	I, II, III	ND	66.7	17	ND	ND
Reardon et al. (35)	Imatinib + hydroxyurea	PDGFR	21	I, II, III	ND	61.9	7	Anemia, constipation, edema, fatigue, hypoalbuminemia, hypophosphatemia, rash, neutropenia, thrombocytopenia	Yes

Important to note, that the CNS NCCN guideline classifies somatostatin analog, as a level 2A category for patients with progressive recurrent meningioma while interferon alpha, sunitinib and bevacizumab, and everolimus combination were given 2B category (6). Category 2A evidence is based on lower-level evidence with uniform NCCN consensus that the intervention is appropriate, while category 2B evidence that is also based on lower level of evidence was only granted experts consensus. These recommendations are not surprising, as scientific literature review indicates that somatostatin analogs including Sandostatin LAR (octreotide) are better tolerated therapies with good efficacy as evidenced by longer PSF and PFS6.

Specifically, phase II clinical trial that included 36 patients with high-grade meningioma who received sunitibin showed efficacy of that treatment based on PFS6 of 42%, median PFS of 5.2 months and median OS of 24.6 months (36). However, considerable toxicity was observed with 1 grade 5, 1 grade 4 and 2 grade 3 intratumoral hemorrhages, 1 grade 4 and 1 grade 3 thrombic microangiopathy attributed to known side effect profile of VEGF inhibitors. By comparison all studies on somatostatin analogs including this report indicate well-tolerability and minimal side effects with diarrhea being the most commonly reported side effect.

The phase II clinical study evaluated efficacy of combination of everolimus and bevacizumab in 17 patients with progressive recurrent meningiomas (WHO tumor grade I,II and III) showed that this regiment was well-tolerated, and produced stable diseases in 88% of patients with median PFS as 22 months, PFS as 69% and median OS of 23.8 months (32). No grade 5 or grade 4 toxicities were reported, but four patients (22%) discontinued treatment due to grade 3 toxicities such as proteinurea, colitis and thrombocytopenia. Important to note, that since the sample size was small, additional work in indicated. In comparison, our study is one of largest studies that included 43 patients providing more conclusive results.

The recently published prospective phase II clinal trial that evaluated the efficacy of combination of everolimus and octreotide reported that stomatitis was the most common grade 3 adverse event, seen in 55% of patients, necessitating the discontinuation of both therapeutics in 1 patient and everolimus in another (24).

A retrospective case series evaluated treatment with interferon alpha for patients with high grade meningiomas that showed progression after surgery, radiotherapy, or prior systemic chemotherapy (37). The study revealed the median PFS12 and PFS6 of 17% without radiographical response and moderate toxicity. Unfortunately, given that overall PFS and PFS6 were below benchmark criteria of PFS of 26% for atypical and malignant meningiomas proposed by the Response Assessment in Neuro-Oncology (RANO) Working Group 2014, it appears to be an unlikely candidate for use for treatment of progressive recurrent meningiomas (38).

Thus, based on available clinical data, Sandostatin LAR (octreotide) should be given consideration for managing patients with progressive and/or recurrent meningiomas. Nevertheless, this was a retrospective study with several limitations, imposed by the type of the study. Comparison of Sandostatin LAR (octreotide) to other therapeutics were hindered, as all the studies have different methodologies, size, patient population, or objectives. In addition, our study did not include a control, and prior reported studies were used for comparison. Furthermore, numerous patients were evaluated years after the initial diagnosis with limited number of patients who were diagnosed based on immunohistochemistry results, hindering further stratification based on molecular profile. Despite the stated limitations, our study is one of the largest retrospective studies that provides rationale and supports further investigation of Sandostatin LAR (octreotide) for the treatment of progressive or recurrent meningiomas. Additional prospective, larger scale randomized trials are needed to validate the effectiveness of Sandostatin LAR (octreotide) in meningioma.

## Data Availability Statement

The datasets for this article are not publicly available because of limitations imposed by the patients confidentially and participant privacy. Requests to access the datasets should be directed to Dr. Maya Hrachova, mayahrachova@gmail.com.

## Ethics Statement

The studies involving human participants were reviewed and approved by University of California Irvine Medical Center. The patients/participants provided their written informed consent to participate in this study.

## Author Contributions

The following authors have contributed significantly to the experimental design (DB, X-TK, GC, and FH), its implementation (MH, EN, BF, MD, X-TK, GC, FH, and DB), or analysis and interpretation of the data (All authors). All authors were involved in the writing of the manuscript at draft and any revision stages and have read and approved the final version.

### Conflict of Interest

The authors declare that the research was conducted in the absence of any commercial or financial relationships that could be construed as a potential conflict of interest.
